# Neutral excitation density-functional theory: an efficient and variational first-principles method for simulating neutral excitations in molecules

**DOI:** 10.1038/s41598-020-65209-4

**Published:** 2020-06-02

**Authors:** Subhayan Roychoudhury, Stefano Sanvito, David D. O’Regan

**Affiliations:** School of Physics, AMBER and CRANN Institute, Trinity College Dublin, The University of Dublin, Dublin 2, Ireland

**Keywords:** Density functional theory, Atomic and molecular interactions with photons, Electronic structure of atoms and molecules

## Abstract

We introduce neutral excitation density-functional theory (XDFT), a computationally light, generally applicable, first-principles technique for calculating neutral electronic excitations. The concept is to generalise constrained density functional theory to free it from any assumptions about the spatial confinement of electrons and holes, but to maintain all the advantages of a variational method. The task of calculating the lowest excited state of a given symmetry is thereby simplified to one of performing a simple, low-cost sequence of coupled DFT calculations. We demonstrate the efficacy of the method by calculating the lowest single-particle singlet and triplet excitation energies in the well-known Thiel molecular test set, with results which are in good agreement with linear-response time-dependent density functional theory (LR-TDDFT). Furthermore, we show that XDFT can successfully capture two-electron excitations, in principle, offering a flexible approach to target specific effects beyond state-of-the-art adiabatic-kernel LR-TDDFT. Overall the method makes optical gaps and electron-hole binding energies readily accessible at a computational cost and scaling comparable to that of standard density functional theory. Owing to its multiple qualities beneficial to high-throughput studies where the optical gap is of particular interest; namely broad applicability, low computational demand, and ease of implementation and automation, XDFT presents as a viable candidate for research within materials discovery and informatics frameworks.

## Introduction

The first-principles calculation of the excited-state energies of quantum systems holds crucial importance for the study of solar cells^1^, organic light emitting diodes^2^, and chromophores in biological systems^3^, to name but a few. With some exceptions, density-functional theory (DFT), the primary *ab initio* workhorse for computing ground state properties^4,5^, typically falls short on such tasks, although efforts are underway to extend the foundation of DFT to excited states^6–11^. The most commonly used first-principles method for calculating excitation energies, at least of finite systems, is linear-response time-dependent density functional theory (LR-TDDFT)^12–14^. However, LR-TDDFT has two significant limitations: its computational cost, which severely limits the size of the systems that it can be used to investigate^15^, and its inability to treat double (two-electron) or higher-order excitations within adiabatic approximations to the exchange-correlation (XC) kernel^16–18^. Such multi-particle excitations and indeed inter-conversions between excitation types are of considerable interest, not alone on fundamental grounds, but for example in the computational development of photovoltaic materials that intrinsically overcome the Shockley-Queisser limit.

Often, it is the lowest excitation energy of a given symmetry that is of principal interest, as this can be used to calculate the electron-hole binding energy. In such cases, state-of-the-art methods that rely on the coupling of *all* excitations, such as real-time TDDFT, are inefficient. These considerations strongly motivate the development of non-perturbative, variational methods based on time-independent DFT for excited states. Ideally, the desired method should inherently capture response to all orders and preserve a favourable computational scaling, while avoiding slowly-converging sums over virtual states.

Over the years, several such first-principles schemes have been developed for calculating neutral excitation energies, such as ensemble DFT^19–21^, restricted open-shell Kohn-Sham DFT^22–25^, constricted variational DFT^26,27^, Δ self-consistent field (ΔSCF) -DFT^28,29^, constrained DFT defined using virtual Kohn-Sham states^30^, and the maximum overlap method^31,32^. These are underpinned by the existence of a variational DFT, with an equivalent non-interacting Kohn-Sham (KS) state, for an individual excited state of interacting electrons^6,7,28^. Each method, including the one introduced here which builds upon the foundation that others have provided, has its relative strengths and weaknesses in terms of computational cost and ease of both implementation and convergence. We refer the reader to ref. ^33^ for a recent review of TDDFT, and to ref. ^26^ for a foundational comparison between TDDFT and DFT-based variational approaches.

In this article, we introduce *neutral excitation DFT* (XDFT), an inexpensive, fully first-principles extension of constrained DFT (cDFT)^34–36^ for calculating neutral excitation energies in finite systems such as molecules and clusters. XDFT simulates an excitation in the KS system by reducing the electronic population of the ground state valence subspace by one electron, while keeping the total number of electrons unchanged. XDFT is quite unlike conventional cDFT in that no prior assumptions are made as to the spatial form of source or drain regions for charge constraint. It captures screening effects at all orders, unlike LR-TDDFT, but retains a single Slater determinant of Kohn-Sham orbitals and so it is readily portable to many standard DFT codes (this transpires to be drawback, as we will see, in the study of singlet excitations, motivating future developments). XDFT scales with the atom count, *N*, as per ground-state DFT, namely as ${\mathscr{O}}({N}^{3})$ when no quantum nearsightedness is exploited (we have implemented it within an ${\mathscr{O}}(N)$ code). This contrasts with methods like TDDFT, which typically scales as ${\mathscr{O}}({N}^{4})$^37^ and the Bethe-Salpeter equation (BSE), which goes as ${\mathscr{O}}({N}^{6})$^38^. In addition, unlike LR-TDDFT and BSE, which can be highly memory intensive when unoccupied states are treated explicitly, XDFT has a memory overhead comparable to that of standard DFT. Crucially, it avoids the calculation of unoccupied ground-state KS orbitals entirely, and we never calculate them here in practice. Therefore, in terms of computational efficiency, XDFT offers significant advantages over the aforementioned existing methods, as long as only the lowest-energy excited state of a given symmetry is of particular interest. XDFT offers ready compatibility with high-throughput frameworks^39^, the study of large-systems, and variational KS methods beyond DFT.

Applying this technique, we calculate the lowest singlet and triplet single excitation energies of a representative set of organic molecules. Here we find a surprisingly good agreement with LR-TDDFT values, notwithstanding the extreme simplicity of our method. We also move straightforwardly beyond single-particle excitations, while still only using a small number of coupled DFT calculations. As we will see, XDFT can accurately reproduce the energies of excited states with a predominantly double-excitation character in the canonical test-bed atom beryllium, which are inaccessible^16–18^ to adiabatic LR-TDDFT.

In the next section, we describe the XDFT formalism. This is followed by a section that explores the connection between XDFT and exact theorems of excited state DFT and outlines the relevant approximations.  The next section  outlines certain important details of the calculation. Results, including single and double excitation energies and difference density are presented in the penultimate section. Finally, we present our conclusions.

## The XDFT Formalism in Brief

A neutral excitation, within the quasiparticle picture, is the promotion of one or more electrons from occupied levels to empty ones, resulting in the creation of bound electron-hole pairs and with consequent energy relaxation due to screening. To simulate this excited state, XDFT searches for the ground-state energy of the same system, but now subject to the extra condition of a given number of electrons with spin $\sigma$, ${N}_{c}^{\sigma }$ being confined to a pre-defined subspace. This constraining condition may be written as

1 $${\rm{Tr}}[{\hat{\rho }}^{\sigma }\hat{{\mathbb{P}}}]={N}_{c}^{\sigma },$$

where ‘Tr’ denotes the trace, ${\hat{\rho }}^{\sigma }$ is the spin-dependent fermionic density operator and $\hat{{\mathbb{P}}}$ is a projection operator onto the desired subspace. In conventional cDFT, the subspace spanned by $\hat{{\mathbb{P}}}$ is a spatial region defined at the researcher’s discretion. If $\hat{{\mathbb{P}}}$ spans two spatial regions with opposite weighting, for example, then one can enforce a charge-separated density configuration for the simulation of charge-transfer excitations^40–44^.

Here we come to the key development of XDFT. In order to access excitations beyond charge-separated states of obvious spatial character, we free cDFT of all human assumptions by defining the subspace in terms of the ground-state KS eigenstates only. In doing so we retain the physical information encoded in the KS eigensystem, which is a key ingredient in LR-TDDFT but discarded in normal cDFT. More technically speaking, in a neutral *N*-electron system XDFT locates the energy of the lowest *M*-electron excited state (*M* may even be non-integer in principle) by confining *N*–*M* electrons within the valence KS subspace of the unconstrained DFT ground-state. This circumvents the need for any prior, empirical specification of subspaces and restores first-principles status. The projector that *defines* XDFT is

2 $$\hat{{\mathbb{P}}}={\hat{\rho }}_{0}=\sum _{i}{f}_{i}|{\psi }_{i}\rangle \langle {\psi }_{i}|,$$

where ${\hat{\rho }}_{0}$ is the ground state density operator, $|{\psi }_{i}\rangle$ is the *i*^th^ KS orbital and ${f}_{i}$ is its occupation number and the sum is over all KS levels. Note that, at zero temperature, ${f}_{i}\mathrm{=1(0)}$ for occupied (unoccupied) levels.

As in conventional cDFT, the ground state of the system subject to the “exciting” constraint in XDFT is found at the stationary point of the functional

3 $$W[\hat{\rho },{V}_{c}]=E[\hat{\rho }]+{V}_{c}({\rm{Tr}}[{\hat{\rho }}^{\sigma }\hat{{\mathbb{P}}}]-{N}_{c}^{\sigma }),$$

where ${V}_{c}$ is a Lagrange multiplier. For a fixed ${V}_{c}$, the second term on the right-hand side serves to modify the ground state potential by adding the term ${V}_{c}\hat{{\mathbb{P}}}$. One then minimises $W[\hat{\rho },{V}_{c}]$ with respect to $\hat{\rho }$, just as $E[\hat{\rho }]$ is minimized in regular DFT. At the *V*_*c*_-dependent minima $W[\hat{\rho },{V}_{c}]$ can be thought of as a function^36^, *W*(*V*_*c*_), of *V*_*c*_. The maxima of *W*(*V*_*c*_) occur at stable states of the constrained system^45^, at which the value of *W* is the excited-state total energy of interest. Once the energy of the first excited state, *W*, is determined by optimising *V*_*c*_, then the lowest excitation (photon absorption) energy, ${E}^{\ast }$, can be evaluated as a total energy difference from the ground state DFT energy, ${E}_{0}$, namely as ${E}^{\ast }=W-{E}_{0}$.

We note that excitations beyond the lowest-energy one of a given spin symmetry can be simulated in XDFT by employing multiple constraints. For example, if the Kohn-Sham valence subspaces of the ground state and the first XDFT excited state are projected onto by ${\hat{{\mathbb{P}}}}_{0}$ and ${\hat{{\mathbb{P}}}}_{1}$, respectively, then the energy of the second excited state can be found by confining $N-1$ electrons within the subspace of ${\hat{{\mathbb{P}}}}_{0}$ using a Lagrange multiplier ${V}_{c}^{1}$ and, separately, confining $N-1$ electrons within the subspace of ${\hat{{\mathbb{P}}}}_{1}$ using a multiplier ${V}_{c}^{2}$. In general, the total-energy of the *I*^th^ excited state system of a given spin symmetry will be found at the stationary point of

4 $$W=E[\hat{\rho }]+\mathop{\sum }\limits_{i}^{I}{V}_{c}^{i}({\rm{Tr}}[\hat{\rho }{\hat{{\mathbb{P}}}}_{i-1}]-(N-1))\mathrm{}.$$

XDFT can be used to simulate combinations of charge and spin excitations. Given a closed-shell ground state, the double ($M=2$) excitations and the triplet single excitation are straightforward to access with a single constraint. These both incur the cost of just two DFT calculations – the ground-state one and the constrained one. In both cases, the electron-promotion constraint can be applied to the sum of the density operators for each spin, and the triplet state can be selected by setting ${m}_{s}=1$. We have found that, for a common test set with different types of chemical bonds, XDFT allows us to directly access the lowest-lying excitations using coupled ground-state calculations. Non-linear response effects such as many-particle excitations are treated on the same footing as linear-response effects. In essence, while it has long been known that ground-state DFT contains the information needed to calculate neutral excitations, and relatively complex methods have been developed to explore it, we show here that constrained Kohn-Sham DFT at a level easily implementable in any normal DFT code provides a variational approach to exploit this. We note in passing that, since the excited state KS wavefunctions are accessible through XDFT, one can potentially use them to calculate approximate oscillator strengths. This is an interesting topic for future investigations.

## Formal Justification of the XDFT Method

XDFT is formally an orbital-dependent DFT, and its energy is separately invariant under arbitrary unitary transformations among the occupied Kohn-Sham orbitals of the ground-state and of the constrained state. The XDFT constraint, which, for simulation of the first excited state, amounts to ejecting one electron from the valence subspace, encodes a well-defined many-particle excitation of the non-interacting Kohn-Sham system. Now, while this certainly does not imply a well-defined excitation of the interacting system, it may provide a good approximation in cases where the non-interacting and interacting wave-functions are similar (modulo unitary transformations) in both the ground and excited states. This is expected to hold well for systems that do not exhibit strong static correlation, where furthermore we may expect a degree of cancellation of error when only looking at the *differences* of the ground and excited-state total energies. To investigate the validity of the XDFT formalism without having to assume equivalence between the Kohn-Sham and the interacting many-particle wave function, in the following, we seek to establish a rigorous connection between the formally exact theorems of excited state DFT and the XDFT method. We start with a very brief discussion of these exact theorems.

### Exact theorems for excited state DFT

Ground-state density-functional theory involves finding the ground-state (GS) density of a system of interacting electrons through the number-conserving minimisation of the Levy constrained search^46^ energy functional, ${E}_{{\rm{LL}}}[\rho ,{v}_{{\rm{ext}}}]$. Such minimisation is with respect to the density, $\rho ({\bf{r}})$, and for a given external potential, ${v}_{{\rm{ext}}}({\bf{r}})$, where

5 $$\begin{array}{c}{E}_{{\rm{L}}{\rm{L}}}[\rho ,{v}_{{\rm{e}}{\rm{x}}{\rm{t}}}]\\ =\int d{\bf{r}}\,{v}_{{\rm{e}}{\rm{x}}{\rm{t}}}({\bf{r}})\rho ({\bf{r}})+\mathop{min}\limits_{\psi \to \rho }\langle \psi |\hat{T}+{\hat{V}}_{ee}|\psi \rangle \\ =\int d{\bf{r}}\,{v}_{{\rm{e}}{\rm{x}}{\rm{t}}}({\bf{r}})\rho ({\bf{r}})+{F}_{{\rm{L}}{\rm{L}}}[\rho ]\,.\end{array}$$

Here, $\hat{T}$ is the electron kinetic energy operator, ${\hat{V}}_{ee}$ is the electron-electron interaction operator, and ${F}_{{\rm{LL}}}[\rho ]$ is the minimum of $\langle \hat{T}+{\hat{V}}_{ee}\rangle$ provided that the state $|\psi \rangle$ produces the density $\rho ({\bf{r}})$. Now, for some external potential ${v}_{{\rm{ext}}}({\bf{r}})$, we may denote the *k*^th^ excited state, energy and density by $|{\psi }_{k}\rangle$, ${E}_{k}$ and ${\rho }_{k}({\bf{r}})$, respectively. Furthermore, we may assume that, for the same or a different external potential ${v{\prime} }_{{\rm{ext}}}({\bf{r}})$, there exists a stationary state $|\psi {\prime} \rangle$ producing the same density ${\rho }_{k}({\bf{r}})$, but such that

6 $$\langle \psi {\prime} |\hat{T}+{\hat{V}}_{ee}|\psi {\prime} \rangle  < \langle {\psi }_{k}|\hat{T}+{\hat{V}}_{ee}|{\psi }_{k}\rangle \mathrm{}.$$

Then, necessarily,

7 $${F}_{{\rm{LL}}}[{\rho }_{k}({\bf{r}})]\ne \langle {\psi }_{k}|\hat{T}+{\hat{V}}_{ee}|{\psi }_{k}\rangle ,$$

and consequently,

8 $${E}_{{\rm{LL}}}[{\rho }_{k},{v}_{{\rm{ext}}}]\ne {E}_{k}\mathrm{}.$$

This leads us to an important result, shown by Perdew and Levy in ref. ^6^, namely that *a stationary excited state will correspond to a local density minimum of* ${E}_{{\rm{LL}}}[\rho ,{v}_{{\rm{ext}}}]$ *if and only if, for any external potential, there is no other stationary state that gives the same density and yields a lower value for* $\langle \hat{T}+{\hat{V}}_{ee}\rangle$.

In an alternate approach to this^7,47^, the *first* excited-state energy of a system subject to an external potential ${v}_{{\rm{ext}}}({\bf{r}})$ can be found by minimising, with respect to density $\rho ({\bf{r}})$, the functional

9 $${E}_{1}[\rho ,{v}_{{\rm{ext}}}]=\int d{\bf{r}}\,{v}_{{\rm{ext}}}({\bf{r}})\rho ({\bf{r}})+{F}_{1}[\rho ,{v}_{{\rm{ext}}}\mathrm{]}.$$

Here, ${F}_{1}[\rho ,{v}_{{\rm{ext}}}]$ is a bifunctional defined as

10 $${F}_{1}[\rho ,{v}_{{\rm{ext}}}]=\mathop{{\rm{\min }}}\limits_{\{\begin{array}{c}\langle \psi |{\psi }_{0}\rangle \mathrm{=0,}\\ \psi \to \rho \end{array}\}}\langle \psi |\hat{T}+{\hat{V}}_{ee}|\psi \rangle ,$$

where the minimisation of $\langle \hat{T}+{\hat{V}}_{ee}\rangle$ is to be performed over states $|\psi \rangle$, which yield the density $\rho ({\bf{r}})$ and are orthonormal to the ground state, $|{\psi }_{0}\rangle$, of the external potential, ${v}_{{\rm{ext}}}({\bf{r}})$. Foundational justifications for numerous time-independent excited-state DFT schemes^48,49^ including $\varDelta$SCF^28^, TOCIA^50^, and OCDFT^51^ have been developed on the basis of this approach.

Remarkably, if the external potential is Coulombic, namely if, for a number of *M* nuclei, it has the form

11 $${v}_{{\rm{ext}}}({\bf{r}})=\mathop{\sum }\limits_{\alpha \mathrm{=1}}^{M}\frac{-{Z}_{\alpha }}{|{\bf{r}}-{{\bf{R}}}_{\alpha }|},$$

where the *α*^th^ nucleus with charge ${Z}_{\alpha }$ is located at ${{\bf{R}}}_{\alpha }$, then two different stationary states in the presence of the same or different external potential cannot have the same density^52^. In other words, the stationary-state density uniquely specifies the stationary state and the external potential. In this situation, as shown in ref. ^52^, one can, in principle, omit the ${v}_{{\rm{ext}}}$ dependence of ${F}_{1}$ and find the first excited state density ${\rho }_{1}({\bf{r}})$ by minimising

12 $${E}_{1}[\rho ,{v}_{{\rm{ext}}}]=\int d{\bf{r}}\,{v}_{{\rm{ext}}}({\bf{r}})\rho ({\bf{r}})+{F}_{1}[\rho ]\mathrm{}.$$

XDFT may be placed in a formal context using this equation, as we now explain.

### The XDFT approximation

Here we show how the XDFT method follows from Eq. (12) with the use of certain well-defined approximations. Let ${v}_{{\rm{KS}}}({\bf{r}})$ be the KS potential and $|{\psi }_{0}^{{\rm{KS}}}\rangle$ be the KS ground state corresponding to an interacting system with an external potential ${v}_{{\rm{ext}}}({\bf{r}})$. Then, assuming representability where required, consider a non-interacting KS like system whose ground state density equals ${\rho }_{1}$, which is that of the first excited state of the interacting system. This system is subject to a local potential,

13 $${v}_{{\rm{KS1}}}({\bf{r}})={v}_{{\rm{ext}}}({\bf{r}})+\int d{\bf{r}}{\boldsymbol{{\prime} }}\frac{\rho ({\bf{r}}{\boldsymbol{{\prime} }})}{|{\bf{r}}-{\bf{r}}{\boldsymbol{{\prime} }}|}+{v}_{{\rm{XC1}}},({\bf{r}})$$

such that the exchange-correlation potential ${v}_{{\rm{XC1}}}({\bf{r}})$ ensures that the correct density is recovered. Unfortunately, ${v}_{{\rm{XC1}}}({\bf{r}})$ is not known to us.

To facilitate the use of available approximations for exchange-correlation, therefore, let us consider a different non-interacting KS-like system such that its lowest-energy stationary state $|{\bar{\psi }}_{0}^{{\rm{KS}}}\rangle$ which satisfies the condition $\langle {\bar{\psi }}_{0}^{{\rm{KS}}}|{\psi }_{0}^{{\rm{KS}}}\rangle =0$, yields ${\rho }_{1}$. For such a non-interacting system, the XC-potential ${\bar{v}}_{{\rm{XC}}}({\bf{r}})$, which generates a KS potential ${\bar{v}}_{{\rm{KS}}}({\bf{r}})={v}_{{\rm{ext}}}({\bf{r}})+\int d{\bf{r}}{\boldsymbol{{\prime} }}\frac{\rho ({\bf{r}}{\boldsymbol{{\prime} }})}{|{\bf{r}}-{\bf{r}}{\boldsymbol{{\prime} }}|}+{\bar{v}}_{{\rm{XC}}}({\bf{r}})$, contrasts with the standard ground-state XC potential, ${v}_{{\rm{XC}}}({\bf{r}})$, which is constructed in such way that the non-interacting ground-state density associated to ${v}_{{\rm{KS}}}({\bf{r}})={v}_{{\rm{ext}}}({\bf{r}})+\int d{\bf{r}}{\boldsymbol{{\prime} }}\frac{\rho ({\bf{r}}{\boldsymbol{{\prime} }})}{|{\bf{r}}-{\bf{r}}{\boldsymbol{{\prime} }}|}+{v}_{{\rm{XC}}}({\bf{r}})$ coincides with the density of the interacting ground state for the external potential, ${v}_{{\rm{ext}}}({\bf{r}})$. We note, using Eq. (12), that ${\bar{v}}_{{\rm{XC}}}({\bf{r}})$ must be a unique functional of the density. We also note that, in contrast with the corresponding object in the treatment presented in ref. ^53^, $|{\bar{\psi }}_{0}^{{\rm{KS}}}\rangle$ is not generally the first excited state of the original, unconstrained KS system since $|{\bar{\psi }}_{0}^{{\rm{KS}}}\rangle$ and $|{\psi }_{0}^{{\rm{KS}}}\rangle$ are stationary states of KS Hamiltonians with different potentials ${\bar{v}}_{{\rm{KS}}}$ and ${v}_{{\rm{KS}}}$.

Using XDFT, we seek to obtain the non-interacting state $|{\bar{\psi }}_{0}^{{\rm{KS}}}\rangle$ that is orthonormal to the non-interacting ground state $|{\psi }_{0}^{{\rm{KS}}}\rangle$, by ejecting a single electron out of the valence subspace, i.e., by creating an electron-hole pair in the KS system. Formally speaking, therefore, the XDFT method effectively amounts to making the central approximation

14 $${\bar{v}}_{{\rm{XC}}}[\rho ]\approx {v}_{{\rm{XC}}}[\rho \mathrm{]}.$$

Variational collapse to the ground state density does not arise in XDFT, in spite of this approximation, since we only search for $|{\bar{\psi }}_{0}^{{\rm{KS}}}\rangle$ that satisfy $\langle {\bar{\psi }}_{0}^{{\rm{KS}}}|{\psi }_{0}^{{\rm{KS}}}\rangle \mathrm{=0}$ and so the ground-state density generated $|{\psi }_{0}^{{\rm{KS}}}\rangle$ is out of bounds. A similar approximation to Eq. (14), in terms of the exchange-correlation energy functional, has been used in OCDFT^51^. We refer the reader in particular to the very informative Table 3 of ref. ^51^, where several properties of various time-independent excited-state DFT approaches are carefully compared. Using the same notation as is used in that Table, the properties of the XDFT method are tabulated in Table 1.

**Table 1:** Using the same notation as is introduced in Table 3 of ref. ^51^, the properties of XDFT from the left-most column report (i) orthonormality with respect to the ground state; (ii) the number of electrons transferred from the occupied to the virtual orbitals; (iii) the orbital space in which the hole orbital belongs; (iv) the orbital space in which the occupied orbitals belong, and (v) whether the method may suffer from variational collapse to the ground state.

Method	${\boldsymbol{\langle }}{\boldsymbol{\Phi }}{\boldsymbol{|}}{\boldsymbol{\Phi }}{\boldsymbol{{\prime} }}{\boldsymbol{\rangle }}{\bf{=0}}$	$\varDelta \rho$	${{\boldsymbol{\phi }}{\boldsymbol{{\prime} }}}_{{\boldsymbol{h}}}{\boldsymbol{\in }}$	${{\boldsymbol{\phi }}{\boldsymbol{{\prime} }}}_{{\boldsymbol{i}}}{\boldsymbol{\in }}$	Φ-collapse
XDFT	yes	1	V	O⊕V	no

### Proof that XDFT encodes the orthonormality of KS Slater determinants

In the following, we prove that the the density generated by XDFT belongs to a non-interacting state that is orthonormal to $|{\psi }_{0}^{{\rm{KS}}}\rangle$. For an *N* electron system, let the excited-state valence orbitals obtained with XDFT be $\{|{\psi }_{i}\rangle \}$ for $i\mathrm{=1,}\ldots \mathrm{.,}N$. Then, since a unitary transformation of orbitals preserves the density, it is sufficient to prove that there is at least one unitary transformation of $\{|{\psi }_{i}\rangle \}$ that produces an “excited electron” orbital, i.e., an orbital that is orthonormal to all of the ground-state valence orbitals that generate $|{\psi }_{0}^{{\rm{KS}}}\rangle$.

Let us define a candidate orbital $|{\psi }^{\ast }\rangle$, with a view to describing the excited-state component of the XDFT KS valence eigensystem, as

15 $$|{\psi }^{\ast }\rangle =\mathop{\sum }\limits_{i\mathrm{=1}}^{N}(\hat{{\mathbb{1}}}-{\hat{\rho }}_{0})|{\psi }_{i}\rangle \mathrm{}.$$

Then, noting that, for zero-temperature fermionic systems the density matrix is idempotent, and so

16 $$\begin{array}{rcl}{(\hat{{\mathbb{1}}}-{\hat{\rho }}_{0})}^{2} & = & \hat{{\mathbb{1}}}-2{\hat{\rho }}_{0}+{\hat{\rho }}_{0}^{2}\\  & = & \hat{{\mathbb{1}}}-{\hat{\rho }}_{0},\end{array}$$

and that the set of orbitals $\{|{\psi }_{i}\rangle \}$ build a density operator $\hat{\rho }$ obeying the XDFT condition ${\rm{Tr}}[\hat{\rho }{\hat{\rho }}_{0}]=N-1$, by definition, we find that $|{\psi }^{\ast }\rangle$ is not necessarily normalised, since

17 $$\begin{array}{rcl}\mathrm{0 < }{\mathcal{C}} & \equiv  & \langle {\psi }^{\ast }|{\psi }^{\ast }\rangle =\mathop{\sum }\limits_{i,j\mathrm{=1}}^{N}\langle {\psi }_{i}|(\hat{{\mathbb{1}}}-{\hat{\rho }}_{0})|{\psi }_{j}\rangle \\  & = & \mathop{\sum }\limits_{i,j\mathrm{=1}}^{N}{\delta }_{ij}-\mathop{\sum }\limits_{i,j=1}^{N}\langle {\psi }_{i}|{\hat{\rho }}_{0}|{\psi }_{j}\rangle \\  & = & N-{\rm{Tr}}[\hat{\rho }{\hat{\rho }}_{0}]-\mathop{\sum }\limits_{i\mathrm{=1},\,{\rm{j}}\ne i}^{N}\langle {\psi }_{{\rm{i}}}|{\hat{\rho }}_{0}|{\psi }_{{\rm{j}}}\rangle \\  & = & 1-2\Re \mathop{\sum }\limits_{i\mathrm{=1,}j < i}^{N}\langle {\psi }_{i}|{\hat{\rho }}_{0}|{\psi }_{j}\rangle \mathrm{}.\end{array}$$

With the latter in hand, let us consider a unitary transformation ${\bf{U}}$ of the orbitals $\{|{\psi }_{i}\rangle \}$ such that

18 $$|{\psi {\prime} }_{j}\rangle =\mathop{\sum }\limits_{i\mathrm{=1}}^{N}{U}_{ij}|{\psi }_{i}\rangle ,\forall \,j=1,\,\ldots .,N,$$

noting that unitarity imposes the requirement that

$$\langle {\psi {\prime} }_{j}|{\psi {\prime} }_{k}\rangle ={\delta }_{jk},\forall .j,k=1,\,\ldots ,\,N.$$

While the right-hand side of Eq. (15) is not the linear expansion of $|{\psi {\prime} }_{N}\rangle$ in terms of the set $\{|{\psi }_{i}\rangle \}$, we know that such a unique linear expansion exists, since $(\hat{{\mathbb{1}}}-{\hat{\rho }}_{0})$ is a projection operator. Thus, in principle, we can uniquely specify a row of ${\bf{U}}$, ${U}_{iN}$ for all $i=1,\ldots .,N$, such that $|{\psi {\prime} }_{N}\rangle =|{\psi }^{\ast }\rangle {C}^{-\mathrm{1/2}}$, simultaneously satisfying the normalisation demand of unitary transformations. The problem of finding any suitable remaining $\{|{\psi {\prime} }_{i}\rangle \}$ boils down to constructing any $(N\times N)$ matrix ${\bf{U}}$ that satisfies the condition for unitarity

19 $$\mathop{\sum }\limits_{k\mathrm{=1}}^{N}{U}_{ki}{U}_{kj}^{\ast }={\delta }_{ij},\,\forall \,i,j=\mathrm{1,}\ldots ,N$$

and whose *N*-th column is uniquely known. Avoiding double counting of equations for $i\ne j$, Eq. (19) is a set of $({N}^{2}+N-\mathrm{2)/2}$ equations, omitting the equation for $i=j=N$ since it contains known terms only. For the $({N}^{2}-N)$ unknowns, this is always solvable (for $N > 2$, solvable with infinite solutions).

Now, recalling that ${\hat{\rho }}_{0}-{\hat{\rho }}_{0}^{2}={\hat{\rho }}_{0}-{\hat{\rho }}_{0}=\hat{0}$, we have

20 $${\hat{\rho }}_{0}|{\psi {\prime} }_{N}\rangle ={\hat{\rho }}_{0}(\hat{{\mathbb{1}}}-{\hat{\rho }}_{0})\mathop{\sum }\limits_{i\mathrm{=1}}^{N}|{\psi }_{i}\rangle {{\mathcal{C}}}^{-\mathrm{1/2}}=|0\rangle ,$$

and therefore $|{\psi {\prime} }_{N}\rangle$ is orthonormal to all of the ground-state valence orbitals. Hence, the Slater determinant constructed from the set of orbitals $\{|{\psi {\prime} }_{i}\rangle \}$ is necessarily orthonormal to the determinant $|{\psi }_{0}^{{\rm{KS}}}\rangle$.

## Methodological Details

### Implementation and parameters

The linear-scaling first-principles code  ONETEP^54^, within which we have implemented the XDFT formalism, variationally optimizes a minimal set of localized, non-orthonormal generalized Wannier Functions (NGWF), expanded in terms of psinc functions^55,56^, to minimize the total energy. ONETEP is equipped with an automated conjugate-gradients method for optimizing the cDFT (or XDFT) Lagrange multiplier^45,57,58^. We have used this, together with the Perdew-Burke-Ernzerhof (PBE) XC functional^59^ to calculate the lowest singlet excitation energies of the 28 closed-shell organic molecules contained in the well-known Thiel set^60^. Our calculations are performed using scalar relativistic norm-conserving pseudopotentials, a plane-wave cutoff energy of 1500 eV and a radius of 14.0 a_0_ for the NGWFs. The Martyna-Tuckerman periodic boundary correction scheme^61^ was used with a parameter of 7.0 *a*_0_. The constrained KS system was found to contain symmetry-protected partial-filling of the degenerate highest occupied state in certain molecules, and so we used finite-temperature ensemble DFT as implemented in  ONETEP^62^ in all cases.

### Multiplet sum method

Unlike the density and energy of the triplet first excited state, it is not straightforward to obtain the singlet counterparts with the XDFT method, since, given a closed-shell ground state, the final state of a singlet single excitation can not be represented by a single Slater determinant (Sd). For the non-interacting KS system, any closed-shell excited state corresponding to $[S=0,\,{m}_{s}=0]$ (with a non-interacting energy ${}^{S=0}{E}_{{m}_{s}=0}^{{\rm{K}}{\rm{S}}}$) or open-shell excited state $[S=1,\,{m}_{s}=0]$ (with ${}^{S=1}{E}_{{m}_{s}=0}^{{\rm{K}}{\rm{S}}}$) can, fortunately, be expressed as a linear combination of the same pair of Kohn-Sham Slater determinants (Sds), within a frozen-orbital treatment. These two Sds, which are not eigenstates of ${\hat{S}}^{2}$, are then degenerate, with a non-interacting energy ${}^{Sd}{E}_{{m}_{s}=0}^{{\rm{K}}{\rm{S}}}$. Invoking the multiplet sum method^33,63,64^, we can thereby express the non-interacting energy of a closed-shell singlet state approximately as

21 $${}^{S=0}{E}_{{m}_{s}=0}^{{\rm{K}}{\rm{S}}}=2{\times }^{Sd}{E}_{{m}_{s}=0}^{{\rm{K}}{\rm{S}}}-{}^{S=1}{E}_{{m}_{s}=0}^{{\rm{K}}{\rm{S}}}.$$

In order to access one of these degenerate Sds that make up the singlet, in practice, we promote one electron by applying the XDFT constraint to one spin channel only, maintaining ${m}_{s}=0$. We note that, for a spin-restricted treatment, since the two Sds are degenerate, each of them has the same energy as the singlet and the triplet state and Eq. (21) is trivially satisfied. However, at this point, we make a final assumption that Eq. (21) may be used to approximately evaluate the energy of the interacting system. Keeping in mind that the three triplet states for ${m}_{s}=-\,\mathrm{1,0,1}$ are degenerate, the energy of the singlet first-excited state can then be approximated as

22 $${E}_{{\rm{s}}}\approx 2{E}_{{\rm{Sd}}}-{E}_{{\rm{t}}},$$

where, ${E}_{{\rm{Sd}}}$ and ${E}_{{\rm{t}}}$ are energies of interacting system obtained from XDFT calculations simulating single excitation while maintaining ${m}_{s}=0$ and ${m}_{s}=1$, respectively. The advantage of Eq. (22) is that it involves only the energies of two single-Sd states that are available using XDFT. Each term on the right hand side of Eq. (22) derives from an interacting system that is obtained from an equivalent unrestricted KS system having the same density and spin density. In passing, we note that the Sd state is sometimes referred to in the literature as a *contaminated* singlet. A formalism using restricted (spin-independent) KS orbitals might offer an energy ${E}_{{\rm{Sd}}}$ that is more appropriate for use with Eq. 22.

### Calculation flowchart

**Figure 1:**
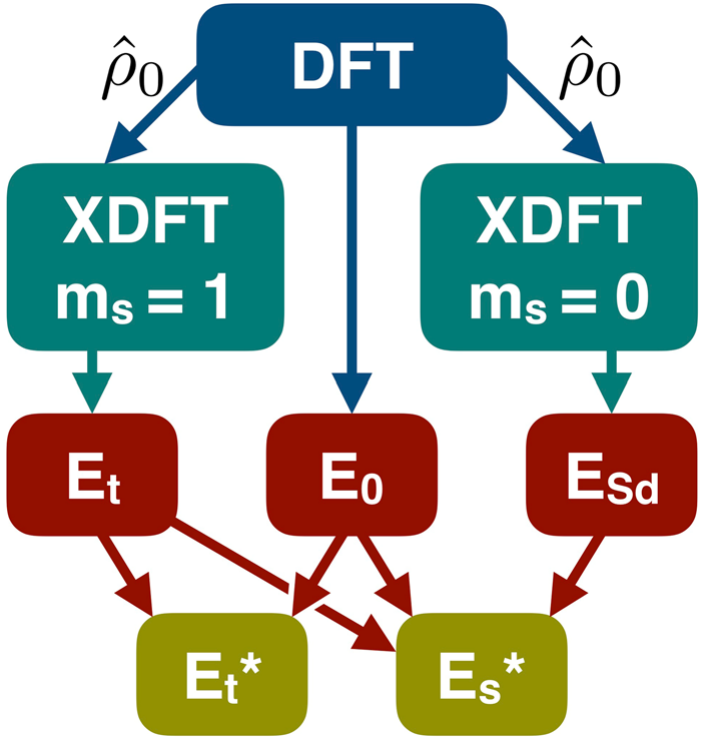
XDFT flowchart for calculating singlet, ${E}_{{\rm{s}}}^{\ast }$, and triplet, ${E}_{{\rm{t}}}^{\ast }$, excitation energies. The ground-state density operator obtained from a DFT calculation, ${\hat{\rho }}_{0}$, is used to define the XDFT constraint. The energies obtained from DFT, ${E}_{0}$, and from XDFT, ${E}_{{\rm{t}}}$ and ${E}_{{\rm{Sd}}}$, are then used to find the excitation energies using Eqs. 22 and 23. Figure created using Apple Keynote v9.1.

The XDFT flowchart involving coupled calculations for finding neutral gaps of finite systems is presented in Fig. 1. In practice, the task of determining the triplet and singlet single excitation energies boils down to calculating total energy differences.

1. First, a standard DFT run is performed to calculate the closed-shell ground-state energy ${E}_{0}$ and density operator ${\hat{\rho }}_{0}$.
2. ${\hat{\rho }}_{0}$ is used to run an XDFT calculation confining $(N-\mathrm{1)}$ electrons to the total valence subspace of the DFT run, with ${m}_{s}=1$ (i.e. fixing a spin moment of $1\,{\mu }_{{\rm{B}}}$). This gives the energy ${E}_{{\rm{t}}}$ of the lowest lying interacting triplet state.
3. Finally, in order to obtain the energy ${E}_{{\rm{Sd}}}$, we run an XDFT calculation confining $(N\mathrm{/2)}-1$ electrons to the spin-up valence subspace of the DFT run while maintaining ${m}_{s}=0$. The singlet first-excited state energy is then obtained from Eq. (22).
4. Ultimately, the triplet and singlet neutral gaps are calculated, respectively, as

23 $${E}_{{\rm{t}}}^{\ast }={E}_{{\rm{t}}}-{E}_{0}\,{\rm{and}}\,{E}_{{\rm{s}}}^{\ast }={E}_{{\rm{s}}}-{E}_{0}\mathrm{}.$$

## Results and Discussion

### Excitation energy of Thiel set molecules

**Figure 2:**
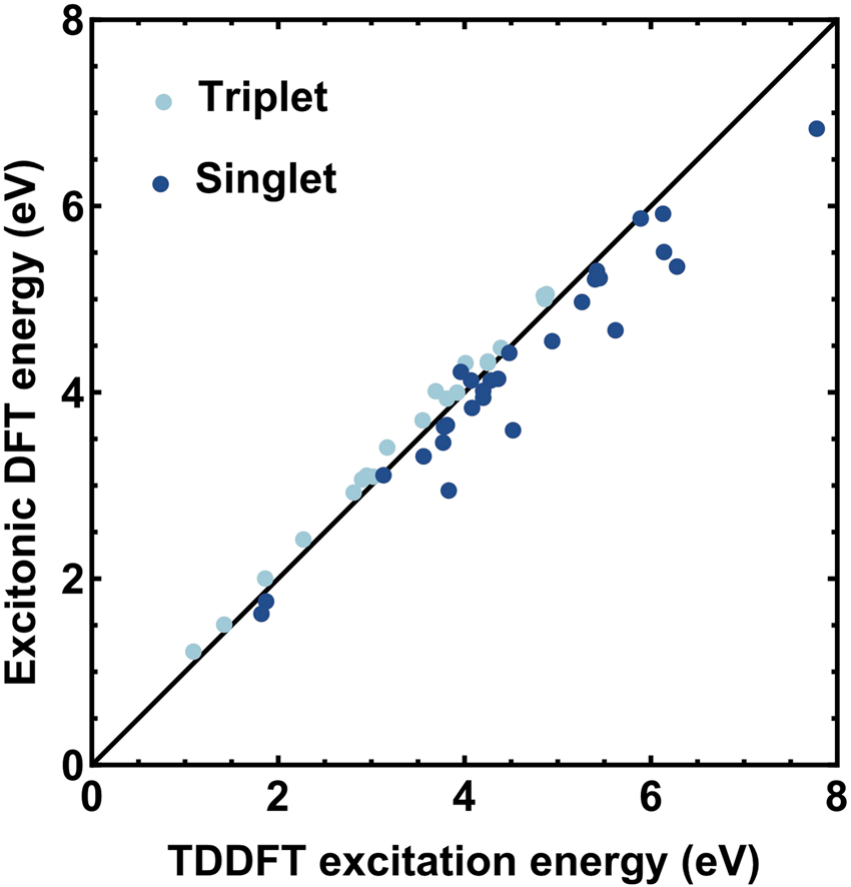
The lowest excitation energies of molecules belonging to the Thiel set^60^ obtained with XDFT and with adiabatic linear-response TDDFT (from refs. ^65,66^). The PBE^59^ XC functional has been used in both cases. The dark and the light dots denote singletand triplet gaps, respectively. The diagonal line indicates perfect agreement between XDFT and TDDFT. Figure created using Wolfram Mathematica v10.3.1.

We have used XDFT to calculate the lowest singlet excitation energies of the 28 closed-shell organic molecules contained in the well-known Thiel set^60^. In Fig. 2, we show a scatter plot of the singlet and triplet excitation energies calculated with XDFT against those obtained with LR-TDDFT and the adiabatic Perdew-Burke-Ernzerhof (PBE) XC functional^59^ in ref. ^65^ (singlets) and ref. ^66^ (triplets). The LR-TDDFT results are broadly in agreement with experimental values (see the supporting information in ref. ^67^). The triplet energies, which do not rely on any additional approximation beyond XDFT such as multiplet sum, show almost perfect agreement with those calculated using ground-state DFT with ${m}_{s}\mathrm{=1}$. This may provide a practical way of validating the XDFT approximations when applied to a new system. The figure also demonstrates that, in spite of the multiplet sum approximation, XDFT yields singlet energies with a remarkably good accuracy, if adiabatic, semi-local LR-TDDFT is taken as a reasonable benchmark. In terms of computational efficiency, we note that, for the representative molecule p-Benzoquinone, an XDFT calculation run on 3 processors for the singlet excitation energy offers an approximate two-fold reduction in computation time compared to its LR-TDDFT counterpart. Unlike linear-scaling LR-TDDFT, XDFT does not require the prior optimization of a defined number of conduction-band orbitals, which is a process that can demand some trial-and-error before well-converged results are obtained. It is to be noted that, as a result of the charge-delocalization error of semi-local XC functionals, XDFT, in its currently-implemented form, is applicable only to finite systems.

### Charge difference density

**Figure 3:**
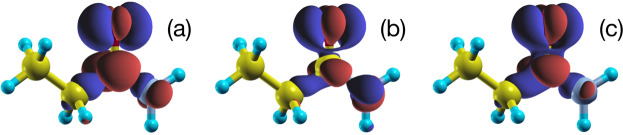
Difference density $\langle {\bf{r}}|(\hat{\rho }-{\hat{\rho }}_{0})|{\bf{r}}\rangle$ for the propanamide molecule, calculated with an isosurface value of ±0.05 eÅ^−3^. Panel (**a**) shows the charge-density difference between the ground-state KS  LUMO and  HOMO orbitals, while (**b**,**c**) show, respectively, the singlet and triplet difference densities generated as the difference between the excited XDFT ($\hat{\rho }$) and ground-state DFT (${\hat{\rho }}_{0}$) densities. The change from (**a**) to (**b**,**c**) is due to electron-hole pair binding and screening, which are captured variationally and at all orders by XDFT. Figure created using XCrysDen v1.6.2^68^ and Apple Keynote v9.1.

The difference between the local part of the constrained density operator, $\hat{\rho }$, and that of the ground state density operator, ${\hat{\rho }}_{0}$, can be viewed as an approximation to the difference density, from which transition dipole moments for example can be calculated. In Fig. 3 we show such plots for a representative molecule of the Thiel set^60^, propanamide. Figure 3(a) shows an approximation to the difference density based on the ground-state KS orbitals, which neglects orbital relaxation and electron-hole binding. Since it captures these effects, the singlet (b) and triplet (c) isosurfaces generated using XDFT (and, in the case of the singlet, the multiplet sum method^33,64^ applied to the total electron densities) reflect a greater degree of difference density localisation than (a). Due to Pauli exclusion, furthermore, the singlet (b) difference density attains a greater spatial localisation than the triplet one (c). Such conclusions are also supported by quantitative analysis of the charge densities.

### Choice of XC functional

The accuracy of results obtained with any DFT-based method is necessarily dependent on the approximate XC-functional used. We have found good agreement between XDFT and LR-TDDFT results for the Thiel set, using the semi-local PBE functional for both methods. This choice of functional is motivated by the fact that, for many DFT and adiabatic-kernel LR-TDDFT calculations, when compared to experimental results, PBE typically offers acceptable accuracy with relatively inexpensive calculations and is therefore a highly popular choice of functional.

However, non-local hybrid XC-functionals containing a fraction of KS exact-exchange typically improves the agreement with experimental results, at the expense of increasing the computational cost. This trend can be seen in Table 2, where we compare experimental singlet excitation energies with XDFT results evaluated using PBE and the B3LYP^74^ hybrid functional for six Thiel set molecules. These molecules are those for which the agreement between the XDFT(PBE) and experimental results is particularly poor (giving a Mean Absolute Error or MAE of 0.85 eV, whereas the MAE is 0.50 eV for the entire Thiel set). The MAE between XDFT and experiment for the six molecules reduces to 0.33 eV using XDFT(B3LYP). It is more probable that this improvement is primarily due to an improved description of the fundamental gap and electron-hole binding, rather than an improvement in the performance of the XDFT or multiplet sum method approximations per se. It is to be noted that, although a hybrid functional improves the energies considerably, the discrepancies resulting from the multiplet sum approximation are present nonetheless. This can be seen by contrasting the MAE (w.r.t. experimental values) of the XDFT results (0.33 eV) with the LR-TDDFT ones (0.08 eV). On the other hand, the fact that, for triplet excitations, the MAE of the XDFT energies is 0.08 eV indicates that the error in the singlet energies arises from the multiplet sum approximation and not from the XDFT approximation *per se*. It seems feasible that this agreement with experiment may be improved further within the XDFT framework, potentially using range-separated hybrid functionals, implicit dielectric screening, zero-point phonon corrections, and a more elaborate treatment of spin contamination that circumvents the multiplet sum method.

**Table 2:** Tabulated values of singlet first excitation energies of six Thiel-set molecules (column 1) for which the agreement between computational and experimental results (column 4) was found to be particularly poor using XDFT using the PBE functional (column 2). The agreement is significantly improved by instead using the B3LYP hybrid functional (column 3). The last row of the table shows the Mean Absolute Error (MAE), which, in our case, is the same as the Mean Unsigned Error (MUE) of the XDFT results with respect to the experimental ones. All energies are in eV, and the multiplet sum method was used to simulate all single-particle singlet excitations.

Molecule	PBE	B3LYP	Experimental
Ethene	6.83	7.28	7.66^69^
p-Benzoquinone	1.75	2.34	2.48^70^
s-Tetrazine	1.62	2.12	2.25^71^
Pyrimidine	3.63	3.94	4.16^72^
Hexatriene	3.59	4.26	4.93^73^
Butadiene	4.67	5.31	5.73^73^
MAE	0.85	0.33	eV

### Simulation of a double excitation

Finally, we explore the ability of XDFT to calculate energies of excitations with strong double (two-electron) character, which are inaccessible by constuction to adiabatic-kernel LR-TDDFT^16–18^. The XDFT method is non-linear, unlike LR-TDDFT, in the sense that its Hartree and XC potentials are calculated self-consistently with the density in the excited state, i.e., not just corrected to first order using the interaction kernel ${\hat{f}}_{{\rm{Hxc}}}$. Thus, XDFT is not limited to single excitations. As a proof of principle, we focus here on one particular excitation of a known, strong double (i.e. two-electron) character, nothing that a more comprehensive study of excitations of more mixed single-double character using XDFT would be necessary to fully establish the range of applicability of the method to such effects. We note, in passing, that XDFT is not restricted to exciting integer numbers of electrons, *M*, particularly when coupled with ensemble DFT. In the benchmark case of atomic beryllium, the first double excitation promotes two electrons from the 2*s* to the 2*p* orbitals^75^. For the Be atom, 1*s* electrons are described by a pseudopotential, rendering the multiplet sum method unnecessary. Consequently, using the ground state valence density operator ${\hat{\rho }}_{0}$ to confine zero electrons to the total valence subspace from a standard ground-state DFT calculation, we can directly access the energies of the lowest lying doubly excited singlet $({}^{0}E_{0}^{\mathrm{(2)}})$ and triplet $({}^{1}E_{1}^{\mathrm{(2)}})$ states with two separate XDFT calculations with ${m}_{s}=0$ and ${m}_{s}=1$ respectively.

**Figure 4:**
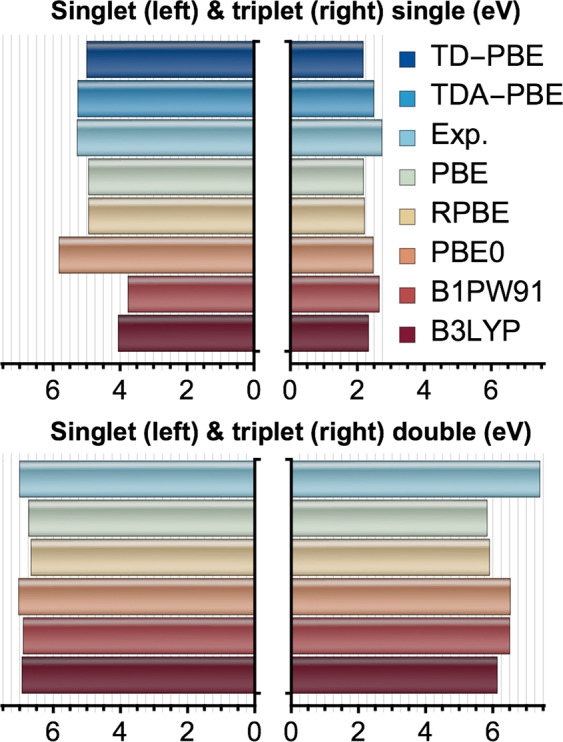
The excitation energies of atomic Be from experiment, linear-response TDDFT (the ONETEP linear-scaling implementation^76,77^), and XDFT. The top panels show single-electron excitation energies and the bottom panels show double (two-electron) excitation energies. The functionals tested were the generalised gradient approximation parameterisations PBE^59^ and RPBE^78^, and the hybrid functionals PBE0^79^, B3LYP^74^, and B1PW91^80^. For the single excitations, we have included the results of linear-response TDDFT calculations within adiabatic PBE, where double excitations are inaccessible. TDA here refers to the same calculations but within the Tamm-Dancoff approximation^81^. We also provide experimental values taken from ref. ^82^. Figure created using Wolfram Mathematica v10.3.1.

In Fig. 4 we plot the single and double excitation energies of Be calculated with semi-local and hybrid XC-functionals. The singlet and triplet double excitation energies were obtained as ${}^{0}\,{E}^{\mathrm{(2)}\ast }={}^{0}\,{E}_{0}^{\mathrm{(2)}}-{E}_{0}$ and ${}^{1}\,{E}^{\mathrm{(2)}\ast }={}^{1}\,{E}_{1}^{\mathrm{(2)}}-{E}_{0}$, respectively. Our results agree well with those calculated with ensemble DFT in ref. ^20^, for all four excitation types. The singlet single-electron PBE excitation energy is also in very close agreement with our own LR-TDDFT(PBE) result, indicating that the multiplet sum method is accurately applicable to this system. We note, however, that while our singlet double excitation energies agree surprisingly well with experimental values, this is much less the case for our triplet double energies. Experimentally, the singlet $2{s}^{2}\to 2{p}^{2}$ excitation is slightly lower in energy than the triplet one, and this has been explained as resulting from a mixing of the singlet double with higher singlet single excitations^82^. Our results would support the opposite conclusion about the mechanism behind this anomalous ordering (singlet below triplet), however, since it is the triplet state which is poorly described by the single-determinant theory. In principle, XDFT is capable of accessing excitations of non-integer electron character (e.g, mixed single and double excitations) with the aid, e.g., of ensemble DFT^62,83^, and this is a promising avenue for future investigation.

## Conclusion

In summary, we introduce the XDFT method for calculating the excited-state energies of finite systems by means of a small number of coupled DFT calculations. The XDFT method, which, with certain approximations, can be arrived upon from the exact theorems of excited state DFT, generalizes constrained DFT, in essence, by removing the necessity for users to pre-define the targeted subspaces. Unlike standard implementations of LR-TDDFT or BSE, no reference is made to unoccupied orbitals. XDFT closely reproduces the LR-TDDFT values for triplet and also, with the help of an additional approximation, in most cases the singlet excitation energies of the Thiel molecular test set. XDFT(B3LYP) offers significantly improved singlet energies with respect to experiment, over XDFT(PBE). Interestingly, however, XDFT can access the energies of double excitations, in principle, effectively circumventing the requirement for non-adiabaticity in LR-TDDFT. We demonstrate this in a successful application to the well-known beryllium test case as a proof of principle.
